# Back from a Predicted Climatic Extinction of an Island Endemic: A Future for the Corsican Nuthatch

**DOI:** 10.1371/journal.pone.0018228

**Published:** 2011-03-25

**Authors:** Morgane Barbet-Massin, Frédéric Jiguet

**Affiliations:** Muséum National d'Histoire Naturelle, UMR 7204 MNHN-CNRS-UPMC, Centre de Recherches sur la Biologie des Populations d'Oiseaux, Paris, France; Instituto de Higiene e Medicina Tropical, Portugal

## Abstract

The Corsican Nuthatch (*Sitta whiteheadi*) is red-listed as vulnerable to extinction by the IUCN because of its endemism, reduced population size, and recent decline. A further cause is the fragmentation and loss of its spatially-restricted favourite habitat, the Corsican pine (*Pinus nigra laricio*) forest. In this study, we aimed at estimating the potential impact of climate change on the distribution of the Corsican Nuthatch using species distribution models. Because this species has a strong trophic association with the Corsican and Maritime pines (*P. nigra laricio and P. pinaster*), we first modelled the current and future potential distribution of both pine species in order to use them as habitat variables when modelling the nuthatch distribution. However, the Corsican pine has suffered large distribution losses in the past centuries due to the development of anthropogenic activities, and is now restricted to mountainous woodland. As a consequence, its realized niche is likely significantly smaller than its fundamental niche, so that a projection of the current distribution under future climatic conditions would produce misleading results. To obtain a predicted pine distribution at closest to the geographic projection of the fundamental niche, we used available information on the current pine distribution associated to information on the persistence of isolated natural pine coppices. While common thresholds (maximizing the sum of sensitivity and specificity) predicted a potential large loss of the Corsican Nuthatch distribution by 2100, the use of more appropriate thresholds aiming at getting closer to the fundamental distribution of the Corsican pine predicted that 98% of the current presence points should remain potentially suitable for the nuthatch and its range could be 10% larger in the future. The habitat of the endemic Corsican Nuthatch is therefore more likely threatened by an increasing frequency and intensity of wildfires or anthropogenic activities than by climate change.

## Introduction

The main causes of current species extinctions are the destruction and fragmentation of habitats, invasion by alien species and climate change [1]. Some of these factors can have amplified consequences on threatened species on islands, which indeed have been highly vulnerable to recent human activities [2]–[6]. Moreover, even though islands generally hold lower species richness than mainland, they exhibit a high level of endemism and are consequently of high conservation concern [7]–[9].

There are few island endemics in Europe, and among birds, the Corsican Nuthatch is the only French endemic. This nuthatch is nearly exclusively confined to mature groves of Corsican pine, a tree taxon also endemic to Corsica, as the bird feeds mainly on the pine seeds [10]. The Corsican Nuthatch is red-listed as vulnerable to extinction by the International Union for the Conservation of Nature [11] because of its endemism, reduced population size (1,557–2,201 territories) [10] and recent decline, and because its favourite habitat, the mature Corsican pine forest, is currently spatially-restricted (less than 16,000 ha) and decreasing in extend because of fires and logging [12]. The range of the nuthatch is however a bit larger because it also sometimes inhabits groves of Maritime pines [13]. Climate change might be a further threat to this island endemic tree, either directly by shifting suitable climatic conditions further up in altitude, or indirectly by increasing the frequency and/or intensity of forest fires.

Species distribution models are increasingly used in many fields of conservation biology, ecology and evolution [14], and offer the opportunity to assess the potential impacts of environmental changes on species distributions [15], [16]. For models to be reliable, variables implemented in the modelling process must effectively delimit and shape the species distribution, either directly or indirectly [17]. Usually models make use of bioclimatic and land use variables, while considering data from other species can improve predictions in case of strong biotic interactions [18]–[22].

In this study, we aimed at estimating the potential impact of climate change on the distribution of the endemic Corsican Nuthatch, using species distribution models in an ensemble forecast framework. Because this species has a strong trophic association with two local pine species [23], we first modelled the current and future potential distribution of the Corsican pine and of the Maritime pine. Future climate projections of pines and of the nuthatch for 2100 were derived from one general circulation model (HADCM3), modelling physics and dynamics of the atmosphere, under three reports on emission scenarios (A1, A2 and B1), reflecting the potential impacts of different assumptions with respect to demographic, socio-economic and technological development on the release of greenhouse gases. Data for these scenarios were those available at a fine spatial scale (30 arc-seconds) from the IPCC fourth assessment [24].

The Corsican Nuthatch and the associated endemic Corsican pine are currently restricted in range to mountainous woodland, and classical assumptions would predict an altitudinal upward shift of both species in response to climate warming. However, the Corsican pine has suffered large distribution losses in the past centuries due to the development of anthropogenic activities such as logging and settling of pastures, orchards and cultures, especially at low altitude where human densities are higher. As a consequence, the realized niche of the pine is likely significantly smaller and restricted to higher elevation than its fundamental niche, so that a projection of the current distribution under future climatic conditions would produce misleading results. In other words, the Corsican pine is probably able to grow and reproduce at low altitude, under hotter climates, where it has been extirpated only by humans developing food and fibre productions. To obtain a predicted pine distribution at closest to the geographic projection of the fundamental niche, we used available information on the current pine distribution associated to information on the persistence of isolated natural pine coppices to produce binary distributions of the endemic tree. We finally compared the predicted changes in the nuthatch predicted distribution under future climate scenarios if modelling its range using climate and the pines, considering current distributions of the pines associated or not with coppice data for the Corsican pine.

## Materials and Methods

### Biological data

**Figure 1:**
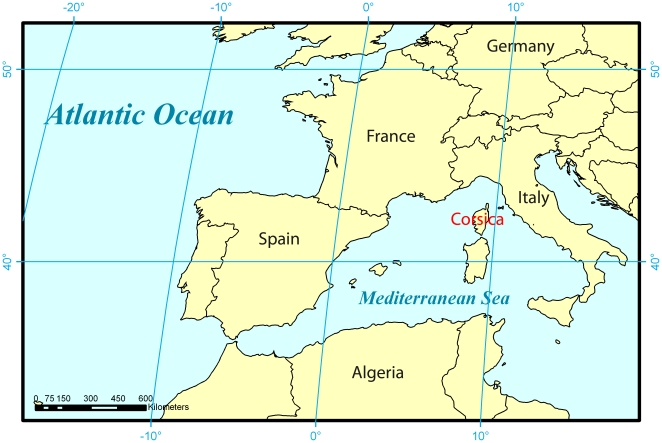
Localisation of the study area.

**Figure 2:**
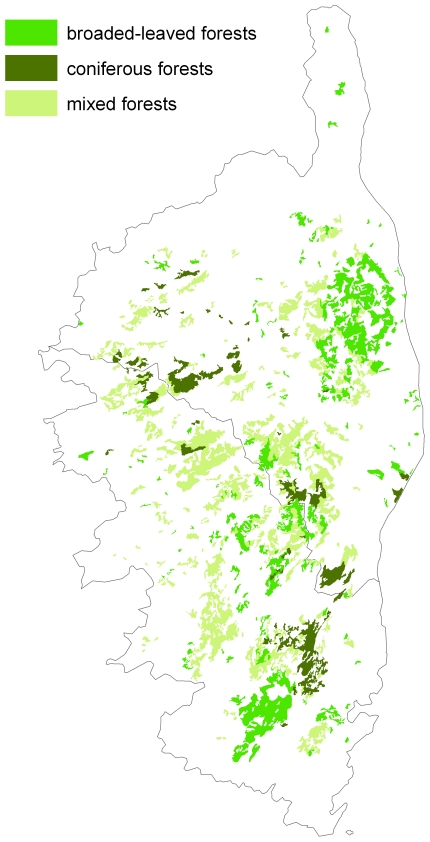
Distribution of forests in Corsica. Light green stands for mixed forests, dark green for coniferous forests and medium green for broad-leaved forests.

**Figure 3:**
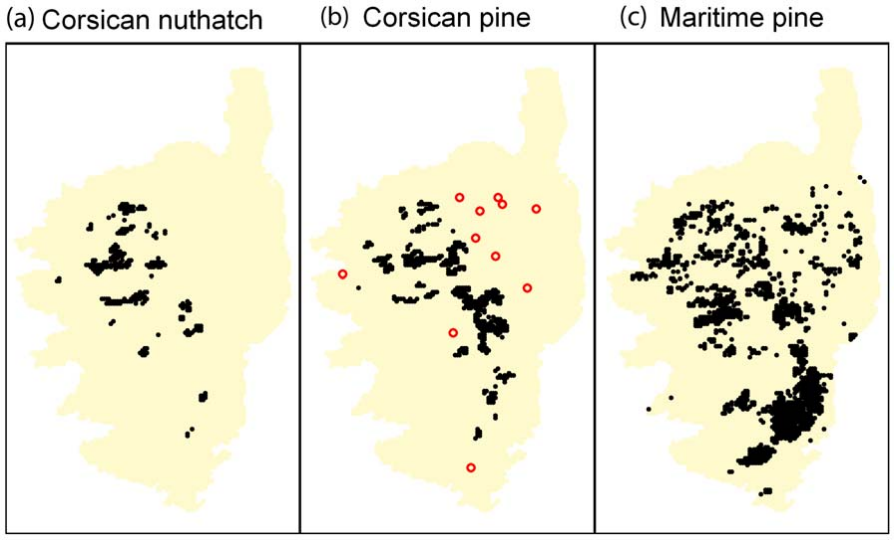
Representation of the data used in the study. (a) Corsican Nuthatch data, (b) Corsican pine data, (c) Maritime pine data. For the Corsican pine red circles represents data from coppices (not used in the niche modelling but to determine further LPT).

The overall spatial extent of Corsica is 8,600 km^2^ with a highest mountain peak reaching 2,700 meters above sea level (Fig. 1). 48 forests were systematically investigated to map nuthatch territories, in the known range of the Corsican Pine, but also in old stands of Maritime Pine. Forests cover 1,416 km^2^ of Corsica (Fig. 2). Overall, this mapping required nearly 20 months of fieldwork by eight different observers who were familiar with the breeding biology and vocalization of the species (see acknowledgements). During the breeding season (March–June) occupied nests were searched for by inspecting trunk cavities. Territorial birds were located mainly by their vocalizations (songs and male-female contact calls). Locations of both were recorded with a GPS (Garmin Summit®, 15 m precision) (Fig. 3).

Distribution ranges of the Corsican and Maritime pines were obtained by digitizing maps published by the Institut Forestier National [25] of France (Fig. 3). Stands of Corsican and Maritime pines are approximately at elevation ranging from 1,000 to 1,800 meters above sea level. For the consideration of thresholds aiming at getting closer to the fundamental distribution of the Corsican pine, we used data from isolated coppices (provided by Jean-Claude Thibault from personal observations).

### Climatic data

Temperature and precipitation are expected to impose direct or indirect constraints on bird distributions [26], while more elaborated climatic variables such as growing degree days and the moisture index have a strong link with the physiology and growth of plant species [27], [28]. For the distribution modelling, we therefore used 10 climatic variables: (i) annual mean temperature, (ii) mean temperature of the warmest month, (iii) mean temperature of the coldest month, (iv) temperature seasonality, (v) annual precipitation, (vi) precipitation of the wettest month, (vii) precipitation of the driest month, (viii) precipitation seasonality, (ix) mean growing degree days and (x) moisture index. The first eight variables were derived from the monthly mean temperatures and precipitations over the period 1961–1990 [29] available at a 30 arc-seconds resolution (for a total of 13459 pixels over Corsica). The last two variables come from the meteorological model Aurelhy [30], based on interpolated measurements at a resolution of 100×100 m. Future climate projections for 2100 were derived from one general circulation model (HADCM3) under three special reports on emission scenarios (A1, A2 and B1), and available from the IPCC fourth assessment [24]. Because the future predictions were only available at a rough scale (3.75°× 2.75°), the anomalies were downscaled to the 30 arc-seconds resolution using a bilinear interpolation and then added to current data.

### Niche modelling

#### Modelling techniques

We used five different niche-based modelling techniques, performed with the BIOMOD computational platform [31]: (1) classification tree analysis (CTA), a classification method running a 50-fold cross-validation to select the best trade-off between the number of leaves of the tree and the explained deviance, (2) artificial neural networks (ANN), a machine learning method, with the mean of three runs used to provide predictions and projections, as each simulation gives slightly different results, (3) mixture discriminant analysis (MDA), a classification method based on mixture models, (4) generalized boosting model (GBM), a machine learning method which combines a boosting algorithm and a regression tree algorithm to construct an ‘ensemble’ of trees, and (5) Random Forest (RF), a machine learning method which is a combination of tree predictors such that each tree depends on the values of a random vector sampled independently and with the same distribution for all trees in the forest. More details about these modelling techniques can be found in Thuiller et al. [31] and in references therein. Such modelling techniques have previously been applied for the purpose of predicting future species distributions [32]–[34].

#### Running and evaluating each modelling technique

Because such techniques require presence and absence data, 5000 pseudo-absences were randomly selected, and because different selections can provide different results, the models were run with 5 different sets of pseudo-absences. For each pseudo-absence run, in order to evaluate the predictive performance of a species distribution model, we used a random subset of 70% of the data to calibrate the model, then used the remaining 30% for evaluation, using a threshold independent method, the area under the relative operating characteristic curve (AUC) [35]. The data splitting approach was then replicated five times from which we calculated the mean AUC of the cross-validation as well as the mean TSS (True Skill Statistic) value [36]. The TSS is the sum of the sensitivity (proportion of actual positives which are correctly identified as such) and the specificity (proportion of negatives which are correctly identified). The final calibration of every model for making predictions uses 100% of available data.

#### Estimating the relative contribution of variables used for niche modelling

Contributions of the variables to the models were obtained with the BIOMOD computer platform: with a permutation procedure, it is possible to extract a measure of relative importance of each variable. Once the models are calibrated, a standard prediction is made. Then, one of the variables is randomized and a new prediction is made. The correlation score between that new prediction and the standard prediction is calculated and is considered to give an estimation of the variable importance in the model.

#### Ensemble forecast

We then used an ensemble forecast technique which aims at accounting for the variability among species distribution models and climate scenarios, in order to get a central tendency [37]. For each pseudo-absence run, the current and future consensus distributions were obtained by calculating the weighted mean of the distributions obtained with the five modelling techniques [38]: the models were ranked according to their predictive performance, and a decay of 1.6 gave the relative importance of the weight (giving respective weights of 0.41, 0.26, 0.16, 0.10 and 0.06). The potential problems raised by Lobo et al. [39] on the use of AUC as a measure of model performance were potentially minor here because AUC was used to select the best models for a given species within a fixed geographical area and using the same pseudo-absences. The final current and future consensus distributions were obtained by calculating the mean across the five pseudo-absence runs. Regarding the future distribution, we calculated the mean distribution between the three available IPCC scenarios.

### Modelling the distribution of pines

Both pine species were modelled using the 10 variables described above (the four temperature variables, the four precipitation variables, the mean growing degree days and the moisture index). Both suitability distributions were then transformed into binary distributions (after applying a threshold) in order to be used as habitat variables for the modelling of the nuthatch distribution. This method leads to several possibilities according to the threshold used. Here, we used three different thresholds. First, we used the threshold maximizing the TSS, a threshold that is commonly used because it produces the most accurate predictions [40]. For the purpose of getting closer to the fundamental niche of the pines, even though over-predicting their current realized distributions, we also used the lowest probability threshold (LPT) [41], whose value is equal to the lowest probability associated with a presence location. Besides, for the Corsican pine, data from current coppices apart from its current distribution in forests were available, so we also used one additional LPT, based on current coppice data (the lowest probability associated with the presence of a coppice).

### Modelling the nuthatch distribution

The Corsican Nuthatch distributions were modelled with the eight climatic variables described above (the four temperature variables and the four precipitation variables) and the two pines distributions. By considering both pines distributions as variables for the modelling of the nuthatch distribution, we assumed a priori no habitat preference of the nuthatch for either one of them even though its preference for the Corsican pine is well established [10]. Nevertheless, all niche modelling techniques used here can weight variables differently accordingly to how the species presences are affected by them.

Because it did not make sense to fit the model for the Corsican Nuthatch with pines distributions that are different to their current distributions, the models were fitted with pines distributions obtained with the TSS threshold, and then projected with current or future data with the pines distributions obtained from the LPTs (and additionally the TSS threshold to compare results from both approaches). Three different current and future distributions of the Corsican Nuthatch were therefore obtained according to the threshold used (TSS, LPT or LPT coppices) to transform the probability distribution of the Corsican pine into a binary distribution (the usual LPT was applied to the Maritime pine while different LPTs were applied to the Corsican pine).

The Corsican Nuthatch distributions were then filtered by applying the LPT of the nuthatch (because all forest with Corsican pines were not studied, so our nuthatch data were probably not representative of the full extent of its distribution), in order to compare current and future ranges obtained with the different methods: all pixels whose suitability was below the threshold were considered outside the distribution, assigning them a zero suitability. An additional distribution was computed, with the pines distributions obtained from the TSS threshold and by further applying the TSS threshold to the nuthatch, in order to evaluate the results of a common modelling technique that would not take into account some specificities of either the Corsican Nuthatch or the pines.

All predicted range sizes were calculated, as well as the mean suitability of each range (as the average of suitability values obtained for all pixels above the threshold) which indicates the mean suitability of the range for this species. Finally, as a way to study how the current known range of the nuthatch is supposed to retract or expand in the future, we calculated the percentage of nuthatch presence points still included in the future distribution, as well as the mean future suitability for these points.

## Results

**Figure 4:**
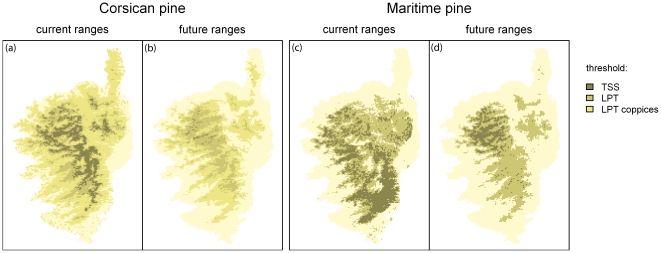
Current and future distributions modelled for the Corsican pine and the Maritime pine. Current (a) and future (b) distributions predicted for the Corsican pine according to the considered threshold. Current (c) and future (d) distributions predicted for the Maritime pine according to the considered threshold.

Both distributions obtained for the pines species have good AUC values of 0.897±0.037 and 0.832±0.060 for the Corsican pine and the Maritime pine, respectively. With thresholds maximizing the TSS, the Corsican pine had a TSS value of 0.694±0.043 and the Maritime pine had a TSS value of 0.520±0.069. Fig. 4 shows the different distributions of the Corsican pine and Maritime pine, obtained with the different thresholds which have been used to model the distribution of the Corsican Nuthatch. The current pine distribution obtained with the TSS threshold is the one used to fit the model, because it is the closest to the data. Current and future distributions obtained with the TSS threshold or the two different LPT were then used for current and future projections of the model. Besides, we can note that for the Corsican pine, the potential future distribution obtained with the LPT is very close to the current distribution obtained with the TSS threshold: almost all current Corsican pine presences are included in the future distribution obtained with the LPT (Fig. 4). Current forests of Corsican pine should therefore not suffer from climate change.

**Figure 5:**
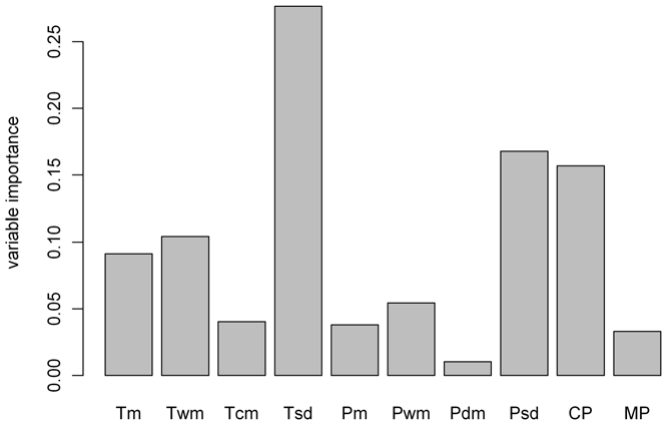
Relative importance of the variables used to model the Corsican Nuthatch distribution. The variable importance was calculated as one minus the correlation between the standard prediction and the prediction where the considered variable was randomized. Tm: annual mean temperature, Twm: mean temperature of the warmest month, Tcm: mean temperature of the coldest month, Tsd: temperature seasonality, Pm: annual precipitation, Pwm: precipitation of the wettest month, Pdm: precipitation of the driest month, Psd: precipitation seasonality, CP: Corsican pine, MP: Maritime pine.

**Figure 6:**
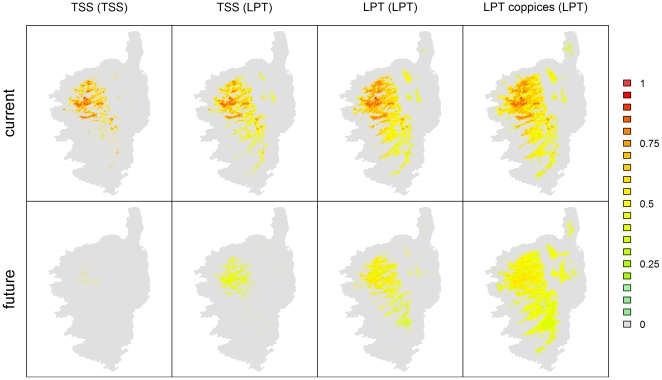
Current and future modelled distributions for the Corsican Nuthatch. The distributions are depicted according to the threshold used to transform the probability distribution of the Corsican pine into a binary distribution, used as one of the variables in the modelling of the Corsican Nuthatch. Only points with suitability above either the LPT or the TSS threshold of the Corsican Nuthatch (in brackets) are represented.

The nuthatch model using the eight climatic variables and both pine distributions had an AUC value of 0.929±0.029 and a TSS value of 0.690±0.034 (when applying the threshold maximizing the TSS). Among the ten variables used to model the nuthatch distribution, three of them turned out to be the main drivers of the nuthatch current distribution: both precipitation and temperature seasonality and the Corsican pine distribution (Fig. 5). Table 1 summarizes range and suitability values obtained with the different models and thresholds (see Fig. 6 for a mapping of the modelling results). The current suitable range of the Corsican Nuthatch obtained with a usual model (both TSS thresholds for the nuthatch and the pines) is 770 km^2^ and is expected to suffer a 97% decrease by 2100 and only 2% of the presence points remain in the future expected range. Results are less pessimistic when applying the LPT to the nuthatch, with a range of 1340 km^2^ expected to decrease by 66%, 50% of the presence points still predicted to be suitable in 2100. The projection of this model with pine binary distributions obtained with a different threshold (getting them closer to their fundamental distribution) leads to larger current ranges (1960 km^2^ with the LPT and 2470 km^2^ with the coppice LPT). In the case of the LPT from forest data (so not considering coppice) for the Corsican pine, the nuthatch range is expected to decrease by 41%, with a decrease in mean suitability as well (from 0.61 to 0.49). Nevertheless, with this model, 71% of the known presence points of the Corsican Nuthatch remain in the future expected range, their mean suitability decreasing from 0.80 to 0.46. Models for which the LPT from coppices data were used give the same results, with a larger modelled current range, and an even larger expected future range, 10% larger than the current range. Nevertheless, the mean suitability of the Corsican Nuthatch is expected to decrease from 0.58 to 0.47, though the differences in values are the smaller we obtained. Besides, with these models, 98% of the current presence points remain included in the future range, their mean suitability decreasing from 0.80 to 0.52.

**Table 1:** Characteristics of the current and future distributions of the Corsican Nuthatch according to the threshold used to transform the pines distributions into binary distributions used as variables for the modelling.

Threshold used for the nuthatch distribution	TSS	LPT		
Threshold used to transform the pine suitability into binary distributions	TSS	TSS	LPT	LPT coppices
Current range (km^2^)	770	1340	1960	2470
Future range (km^2^)	26	455	1150	2740
Current mean suitability	0.68	0.59	0.61	0.58
Future mean suitability	0.58	0.43	0.49	0.47
Proportion of presence points included in the future distribution	0.02	0.50	0.71	0.98
Mean current suitability of presence points	0.79	0.79	0.80	0.80
Mean future suitability of the presences points	0.35	0.35	0.46	0.52

(TSS: True Skill Statistic, LPT: Lowest Probability Threshold).

## Discussion

The distribution models presented here confirm that the distribution of the endemic Corsican Nuthatch is mainly determined by the presence and distribution of the Corsican pine (Fig. 5), as suggested by previous ecological studies [10]. The potential impacts of climate change on the nuthatch distribution will therefore also result from climate change impacts on the Corsican pine. This explained the similarities between future predictions for the Corsican Nuthatch and the Corsican pine. The large differences for the current and future distributions of the Corsican pine depended on the threshold that we used to produce a binary distribution of the pines (see Fig. 4). Choosing an appropriate threshold was therefore very important, even if it often remains difficult and arbitrary [35], [42], [43]. The threshold has to be adapted to available sample size, and to study goals [41]. We could have used the continuous climatic suitability value of the pines distribution in the nuthatch models, but it would not have been possible to disentangle the current observed pine range from potential range closer to the fundamental distribution. Indeed, the distribution of high suitability values was biased towards the realized niche of the species: areas potentially highly suitable for the Corsican Nuthatch because included in its fundamental niche but absent from its realized niche were attributed low suitability values. Such bias would have probably leaded to results similar to the ones obtained with TSS thresholds for pines distribution and the nuthatch distribution.

A classical ensemble forecast modelling to predict the future distribution of the Corsican Nuthatch would have been to use the pine ranges, obtained with TSS thresholds, as variables associated with climatic variables, and to apply a TSS threshold to the obtained suitability value for the bird. In such conditions, the range of the Corsican Nuthatch was predicted to collapse before 2100 (Table 1). By considering that the current distribution of the Corsican Nuthatch was clearly not fulfilling the fundamental niche of the species, we opted for applying a more conservative threshold when producing binary distributions, namely the lowest presence threshold. This threshold designs as presences all sites where the suitability is at least as high as the lowest value associated with any known presence data. Applying such a threshold to the Corsican Nuthatch distribution, but keeping a TSS threshold for mapping the pine ranges, we first predicted that two thirds of the current bird range should be lost because of climate change by 2100, and that the known territories should be four times less suitable. However, current and future predictions for the nuthatch varied largely according to the threshold applied to obtain a binary distribution for the Corsican pine. The realized niche of the Corsican pine was very likely different from its fundamental niche, because of past and ongoing anthropogenic pressures on lowland habitats associated with the development of human societies, and a Lowest Presence Threshold seemed more appropriate too. Indeed, the presence of Corsican pine coppices at low elevation and far from the current mountainous forest distribution of the pine testifies that this taxon is currently distributed – as forests of mature trees - only in a portion of the species' suitable bioclimatic niche. The use of a Corsican pine range that was supposed to be closer to the geographic projection of its fundamental niche (using Lower Presence Thresholds) obviously led to current nuthatch distributions being estimated as larger than the actual range, reflecting the potential bioclimatic distribution of the bird in absence of any human extirpation of the pine.

Considering these models, the actual range of the nuthatch will still be included in its fundamental niche in 2100, and no major changes are expected in the bird distribution. Indeed, we predict that 98% of the known current nuthatch territories should remain suitable by 2100, instead of the 50% obtained with a Corsican pine distribution closer to its realized distribution. Besides, the mean suitability value for these territories was expected to decrease by 35% instead of a 55% decrease, meaning that the population size could possibly decrease even if the range would not contract, but not as much as first expected. Nevertheless, climate change was not expected to be a major direct threat towards the Corsican Nuthatch, because ongoing climate change should not put the current distribution of the Corsican pine outside of its fundamental habitat bioclimatic requirements by 2100. Besides, land use per se was not considered as downscaled future scenarios were not available which could lead to significant over-prediction of suitable habitat if agriculture or urbanization were to gain ground.

Extinction risk of the Corsican Nuthatch during the 21^st^ century should therefore mainly result from habitat changes, mainly logging and wildfires. Because fires are increasing with anthropogenic activities [44] and because wildfires are expected to increase in frequency and intensity with climate change [45], their potential future impacts should be considered seriously. In case of severe fires, the vegetation grows back after being burnt but it takes a century before a pine habitat could grow and become suitable again for the Corsican Nuthatch, which only occupies mature pine groves where trees are at least 100 year old [10]. Conservation efforts should therefore focus on careful planning of the Corsican pine forestry and on actions aiming at reducing the frequency and impact of forest fires. The Corsican pine forest is already considered as a priority habitat by the Council Directive 92/43/EEC (on the Conservation of natural habitats and of wild fauna and flora) adopted by the European Union directive in 1992, and the Corsican Nuthatch is listed in the Appendix 1 of the Council Directive 79/409/EEC on the conservation of wild birds adopted by the European Union in 1979, with dedicated protected and managed areas within the Natura2000 network. Special Protection Areas designed in Corsica include a significant part of the nuthatch population, though only c.30%, and should be managed according to habitat requirements of the bird through appropriate forestry plans. Maintaining old mature pines within large continuous forest patches will be crucial, while post-fire logging should also be adapted. Salvage logging has been encouraged in Corsica during recent years, and a burned stand should not be clear-cut when at least one pine has less than 2.5m of crown burned [23].

Our study intended to explore the potential direct effects of ongoing climate changes, in particular through a potential upward altitudinal shift in the distribution of its habitat. As the actual Corsican pine distribution apparently result from extirpation by human activities at low elevation, the pine is predicted to safely face climate change during the 21^st^ century, and should maintain its mountainous range, so that the nuthatch distribution should not directly suffer much from climate change too. The main threat for the endemic Corsican Nuthatch therefore remains the destruction and fragmentation of mature Corsican pine forests, potentially dependent on indirect effects of climate change beyond the simple bioclimatic components. Indeed, an increased frequency and intensity of droughts could cause increased impacts of wildfires on Corsican forests, impairing the future of the endemic bird. Further modelling developments should try to include such potential impacts of climate change on forest fires to consider uncertainties in the future distribution of the Corsican pine.

## References

[1] Diamond J, Press OU (1989). Overview of recent extinctions.. Conservation of the Twenty-First Century..

[2] Johnson TH, Stattersfield AJ (1990). A GLOBAL REVIEW OF ISLAND ENDEMIC BIRDS.. Ibis.

[3] Cronk QCB (1997). Islands: Stability, diversity, conservation.. Biodiversity and Conservation.

[4] Sadler JP (1999). Biodiversity on oceanic islands: a palaeoecological assessment.. Journal of Biogeography.

[5] Blackburn TM, Cassey P, Duncan RP, Evans KL, Gaston KJ (2004). Avian extinction and mammalian introductions on oceanic islands.. Science.

[6] Sodhi NS, Liow LH, Bazzaz FA (2004). Avian extinctions from tropical and subtropical forests.. Annual Review of Ecology Evolution and Systematics.

[7] Myers N, Mittermeier RA, Mittermeier CG, da Fonseca GAB, Kent J (2000). Biodiversity hotspots for conservation priorities.. Nature.

[8] Kreft H, Jetz W, Mutke J, Kier G, Barthlott W (2008). Global diversity of island floras from a macroecological perspective.. Ecology Letters.

[9] Caujape-Castells J, Tye A, Crawford DJ, Santos-Guerra A, Sakai A (2010). Conservation of oceanic island floras: Present and future global challenges.. Perspectives in Plant Ecology Evolution and Systematics.

[10] Thibault JC, Prodon R, Villard P, Seguin JF (2006). Habitat requirements and foraging behaviour of the Corsican nuthatch Sitta whiteheadi.. Journal of Avian Biology.

[11] IUCN (2010). Red List of Threatened Species.. http://www.iucnredlist.org.

[12] Thibault JC, Prodon R, Moneglia P (2004). Estimating the impact of the fires of summer 2000 on the number of a threatened endemic bird: the Corsican Nuthatch (*Sitta whiteheadi*).. Ecologia mediterranea.

[13] Thibault JC, Hacquemand D, Moneglia P, Pellegrini H, Prodon R Distribution and population size of the Corsican Nuthatch (*Sitta whiteheadi*)..

[14] Guisan A, Thuiller W (2005). Predicting species distribution: offering more than simple habitat models.. Ecology Letters.

[15] Thomas CD, Cameron A, Green RE, Bakkenes M, Beaumont LJ (2004). Extinction risk from climate change.. Nature.

[16] Thuiller W, Lavorel S, Araujo MB, Sykes MT, Prentice IC (2005). Climate change threats to plant diversity in Europe.. Proceedings of the National Academy of Sciences of the United States of America.

[17] Araujo MB, Thuiller W, Yoccoz NG (2009). Reopening the climate envelope reveals macroscale associations with climate in European birds.. Proceedings of the National Academy of Sciences of the United States of America.

[18] Araujo MB, Luoto M (2007). The importance of biotic interactions for modelling species distributions under climate change.. Global Ecology and Biogeography.

[19] Brooker RW, Travis JMJ, Clark EJ, Dytham C (2007). Modelling species' range shifts in a changing climate: The impacts of biotic interactions, dispersal distance and the rate of climate change.. J Theor Biol.

[20] Heikkinen RK, Luoto M, Virkkala R, Pearson RG, Korber JH (2007). Biotic interactions improve prediction of boreal bird distributions at macro-scales.. Global Ecology and Biogeography.

[21] Preston K, Rotenberry JT, Redak RA, Allen MF (2008). Habitat shifts of endangered species under altered climate conditions: importance of biotic interactions.. Global Change Biology.

[22] Van der Putten WH, Macel M, Visser ME (2010). Predicting species distribution and abundance responses to climate change: why it is essential to include biotic interactions across trophic levels.. Philos Trans R Soc B-Biol Sci.

[23] Moneglia P, Besnard A, Thibault JC, Prodon R (2009). Habitat selection of the Corsican nuthatch (*Sitta whiteheadi*) after a fire.. Journal of Ornithology.

[24] IPCC (2007). Climate change 2007: impacts, adaptation, and vulnerability..

[25] IFN Institut Forestier National.. http://www.ifn.fr/.

[26] Root T (1988). ENVIRONMENTAL-FACTORS ASSOCIATED WITH AVIAN DISTRIBUTIONAL BOUNDARIES.. Journal of Biogeography.

[27] Bartlein PJ, Prentice IC, Webb T (1986). CLIMATIC RESPONSE SURFACES FROM POLLEN DATA FOR SOME EASTERN NORTH-AMERICAN TAXA.. Journal of Biogeography.

[28] Prentice IC, Cramer W, Harrison SP, Leemans R, Monserud RA (1992). A GLOBAL BIOME MODEL BASED ON PLANT PHYSIOLOGY AND DOMINANCE, SOIL PROPERTIES AND CLIMATE.. Journal of Biogeography.

[29] Worldclim Global Climate Data.. http://www.worldclim.org/.

[30] Benichou P, Le Breton O (1987). Prise en compte de la topograhie pour la cartographie de champs pluviométriques statistiques : la méthode AURELHY. ‘Agrométéorologie des régions de moyenne montagne’, Toulouse, 16-17 avril 1986. Ed. INRA, Paris (Les Colloques de l'INRA, n° 39).

[31] Thuiller W, Lafourcade B, Engler R, Araujo MB (2009). BIOMOD - a platform for ensemble forecasting of species distributions.. Ecography.

[32] Thuiller W (2004). Patterns and uncertainties of species' range shifts under climate change.. Global Change Biology.

[33] Barbet-Massin M, Walther BA, Thuiller W, Rahbek C, Jiguet F (2009). Potential impacts of climate change on the winter distribution of Afro-Palaearctic migrant passerines.. Biology Letters.

[34] Coetzee BWT, Robertson MP, Erasmus BFN, van Rensburg BJ, Thuiller W (2009). Ensemble models predict Important Bird Areas in southern Africa will become less effective for conserving endemic birds under climate change.. Global Ecology and Biogeography.

[35] Fielding AH, Bell JF (1997). A review of methods for the assessment of prediction errors in conservation presence/absence models.. Environmental Conservation.

[36] Allouche O, Tsoar A, Kadmon R (2006). Assessing the accuracy of species distribution models: prevalence, kappa and the true skill statistic (TSS).. Journal of Applied Ecology.

[37] Araujo MB, New M (2007). Ensemble forecasting of species distributions.. Trends in Ecology & Evolution.

[38] Marmion M, Parviainen M, Luoto M, Heikkinen RK, Thuiller W (2009). Evaluation of consensus methods in predictive species distribution modelling.. Diversity and Distributions.

[39] Lobo JM, Jimenez-Valverde A, Real R (2008). AUC: a misleading measure of the performance of predictive distribution models.. Global Ecology and Biogeography.

[40] Jimenez-Valverde A, Lobo JM (2007). Threshold criteria for conversion of probability of species presence to either-or presence-absence.. Acta Oecologica-International Journal of Ecology.

[41] Pearson RG, Raxworthy CJ, Nakamura M, Peterson AT (2007). Predicting species distributions from small numbers of occurrence records: a test case using cryptic geckos in Madagascar.. Journal of Biogeography.

[42] Manel S, Williams HC, Ormerod SJ (2001). Evaluating presence-absence models in ecology: the need to account for prevalence.. Journal of Applied Ecology.

[43] Liu CR, Berry PM, Dawson TP, Pearson RG (2005). Selecting thresholds of occurrence in the prediction of species distributions.. Ecography.

[44] Syphard AD, Radeloff VC, Hawbaker TJ, Stewart SI (2009). Conservation Threats Due to Human-Caused Increases in Fire Frequency in Mediterranean-Climate Ecosystems.. Conservation Biology.

[45] Brotons L, Jiguet F, Press OU (2010). Climate change and bird communitites.. Effects of climate change on birds..

